# Investigations of Fused Deposition Modeling for Perovskite Active Solar Cells

**DOI:** 10.3390/polym14020317

**Published:** 2022-01-13

**Authors:** Leland Weiss, Tyler Sonsalla

**Affiliations:** Institute for Micromanufacturing, College of Engineering and Science, Louisiana Tech University Ruston, Ruston, LA 71272, USA; tjs041@latech.edu

**Keywords:** micro, perovskite, solar cell, 3D printing, fused deposition modeling

## Abstract

The advent of Fused Deposition Modeling (FDM; or 3D printing) has significantly changed the way many products are designed and built. It has even opened opportunities to fabricate new products on-site and on-demand. In addition, parallel efforts that introduce new materials into the FDM process have seen great advances as well. New additives have been demonstrably utilized to achieve thermal, electrical, and structural property improvements. This combination of fabrication flexibility and material additives make FDM an ideal candidate for investigation of perovskite materials in new solar cell efforts. In this work, we fabricate and characterize a perovskite-based solar cell polymer designed for the FDM fabrication processes. Perovskite solar cells have garnered major research interest since their discovery in 2009. Perovskites, specifically methylammonium lead iodide, offer beneficial properties to solar cell fabrication such as long minority charge carrier distance, high light absorption, and simple fabrication methods. Despite the great potential of these materials, however, stability remains an issue in solar cell utilization as the material degrades under ultraviolet light, exposure to oxygen and water, as well as increased temperatures. To mitigate degradation, different fabrication methods have been utilized. Additionally, multiple groups have utilized encapsulation methods post-fabrication and in situ solution processed integration of polymer materials into the solar cell to prevent degradation. In this paper, we leverage the unique ability of FDM to encapsulate perovskite materials and yield a MAPbI_3_-PCL solar material as the active layer for solar cell use. In this manner, increased ability to resist UV light degradation and material stability from other environmental factors can be achieved. This study provides characterization of the material via multiple techniques like SEM (Scanning Electron Microscopy) and XRD (X-ray Diffraction) as well as absorbance, transmittance, and photocurrent response. Investigations of processing on perovskite degradation as well as initial solar simulated response are recorded. Unique aspects of the resulting material and process are noted including improved performance with increased operating temperature. Increased electron–hole pair generation is observed for 200 μm FDM-printed PCL film, achieving a 45% reduction in resistance under peak incident flux of 590 W/m${}^{2}$ with the addition of MAPbl_3_. This work establishes insight into the use of FDM for full solar cell fabrication and points to the next steps of research and development in this growing field.

## 1. Introduction

Over the course of the past decade, significant changes have occurred in the field of electricity production [1]. Specifically, while the use of traditional fuel sources like coal have diminished, there continues to be a rise in the use of so-called “green” energy sources like solar and wind. It is projected that a continued move from traditional, fossil fuel-based energy sources to renewable sources will continue in the decades ahead. Solar energy power production is poised to be increasingly important among the renewable energy sources between now and 2050 [1]. To support new growth in solar energy production, new methods and materials are needed. Fortunately, there have also been significant advances in unique materials and new methods of manufacture. Fused Deposition Modeling (FDM), or 3D printing, is one such advance.

Given this growing interest in solar-based energy and expanding capabilities of FDM, we investigate new FDM processes and materials that allow the fabrication of 3D printed solar cell active layers. There is inherent promise in the approach given that FDM-based devices are no longer constrained to planar type structures, but may be designed and fabricated for unique spaces, shapes and applications well beyond traditional manufacturing constraints. As an example, a curved-radius active layer will provide improved incidence angles with solar input as the day progresses and the sun moves across the sky. FDM also allows future devices to be fabricated on-site and on-demand in situations where traditional shipping of completed devices is more difficult. This could have significant application in non-terrestrial settings [2,3].

Perovskite solar cells, first discovered in 2009, have quickly achieved efficiencies of over 20% utilizing simple solution-based fabrication methods [4], potentially providing a cost-effective alternative to traditional solar cell technologies. However, perovskite solar cells have also shown stability issues when exposed to ambient atmosphere. It has been shown that UV light, continuous light, oxygen, water, and temperature can affect the stability of perovskite solar cells [5,6,7]. For these reasons, various methods have been utilized during fabrication and post-fabrication to prolong stability. For example, the use of various epoxies and desiccant have been popular methods in post-fabrication [8,9,10]. However, these methods increase the fabrication complexity which ultimately effects scalability to commercial application. Other groups have utilized different chemical pathways (i.e., antisolvents, etc.) to extend the stability and fabricate perovskite solar cells in high humidity environments [11,12,13]. Other efforts have used different electron and hole transport layer combinations to extend the stability of perovskite solar cells in ambient conditions [4,14]. However, commonly utilized electron and hole transport materials can be cost prohibitive for larger scale application (>$300 per gram).

In this current effort, the general approach employed to achieve these unique devices encapsulates perovskite materials for extrusion as polymer-composite filaments through a heated nozzle in a layer-by-layer FDM process. This builds on our prior work and allows the incorporation and utilization of various polymer-based materials with unique, entrained additives [15].

Over the past several years, multiple investigations have undertaken the general challenge of three-dimensionally printed perovskite materials. Beyond the general advantages of FDM noted previously, there are several perovskite-specific advantages to this approach. First, FDM processes utilizing composite filaments have been previously demonstrated to align filler particles upon printing [15]. This improves thermal and electrical properties, which in the context of a solar cell can improve charge extraction and performance. Second, the FDM process integrates a polymer barrier around filler particles, which can protect the particles from ambient conditions (i.e., oxygen and water). In this manner, the long-term stability issues related to perovskite solar cells can be naturally addressed via the fabrication process itself.

Many of the FDM-oriented approaches have focused on uses like photoelectric sensing [16] or light production [17,18,19]. Zhao et al. advanced the general formation of perovskite crystals from disordered to ordered via electric field, producing a more orderly crystal deposition for use in a photosensor with high sensitivity, high stability, and good response [16]. Tai et al. investigated several thermoplastic polymers as protective coatings for perovskite materials and incorporated the effort into light-emitting diode (LED) devices with various emission wavelengths. It was further noted that there was fluorescent behavior of some of the printed structures resulting from this effort [17]. Others have focused on photo-luminescent materials as structural materials [18]. Qaid et al. have investigated the opportunity for light amplification via the alternate CsPbBr_3_ perovskite material while encapsulating in PMMA polymers [20].

In this work, we fabricate and evaluate MAPbI_3_ perovskite material, suitable for FDM printing application and end use as the photo-active layer in solar cell constructions. Fabrication that produces the polymer-encapsulated printable material and full characterization is presented, followed by initial testing of photocurrent operational results in a 3D-printed test piece. This effort sets the foundation for efforts that incorporate the material into a fully realized solar cell.

## 2. Experimental Methods

To achieve a prototype FDM perovskite active layer, processing, characterization, and printing of the required materials was needed. Material was characterized via several processes including SEM (Scanning Electron Microscopy), XRD (X-ray Diffraction), and photocurrent analysis. This section reviews the fabrication processing as well as the test and characterization steps for the fabricated structures and test pieces.

### 2.1. Materials and Fabrication

#### 2.1.1. Perovskite Material Fabrication

This section reviews the material fabrication steps necessary for the creation of the encapsulated perovskite solar material, ready for application as an FDM material. The fabrication of perovskite microcrystals was accomplished utilizing a previously published procedure by Johansson et al. [12]. Methylammonium iodide (MAI) was mixed in isopropyl alcohol until the crystals were completely dissolved. After the crystals were dissolved, lead iodide (PbI_2_) was added in at a 1:1 molar ratio. The resulting solution had a concentration of 0.69 M. After addition of PbI_2_, the solution transparency changed from clear to black signifying the formation of MAPbI_3_.

After formation of the perovskite, 1 wt.% of 3-(2-aminoethylamino) propy-ldimethoxy-methylsilane (i.e., coupling agent (LICA-38)) was added and allowed to stir for 5 min before the addition of polycaprolactone (PCL). PCL was selected as it has a low melting temperature of about 60 °C and performs well as a water/oxygen barrier [21,22,23]. The coupling agent was utilized in order to create a voidless interface between the PCL and MAPbI_3_ crystals. The agent did not degrade the perovskite during preliminary lab testing. Additionally, the printability of filaments was increased with the addition of coupling agent.

**Figure 1:**
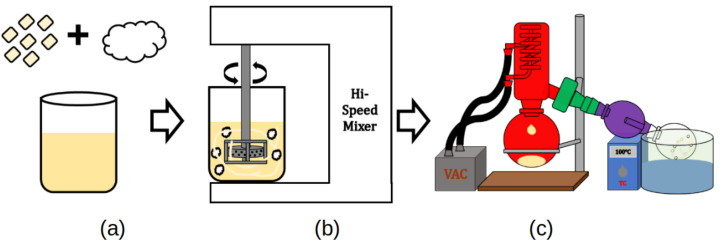
Perovskite composite fabrication process: (**a**) Combining PCL and MAPbI_3_-PCL mixture. (**b**) High-speed mixing. (**c**) Rotary evaporator use to remove IPA.

PCL was added to the mixture to create a 50 wt.% MAPbI_3_-PCL mixture. A 50 wt.% concentration of MAPbI_3_ was selected as it allowed the filament to contain the maximum amount of perovskite particles, while still providing the filament with the ability to be extruded/printed at low temperatures (<100 °C) which prevented degradation. The components were stirred for another 5 min before the solution was added to a recovery flask and IPA was removed from the mixture via rotary evaporator. Figure 1 graphically shows the process.

Following evaporation, the MAPbI_3_-PCL mixture was placed in a Heraeus Instruments Vacutherm oven equipped with a Fisher Scientific Maxima C vacuum pump (delivering pressure less than 1 × $10^{-4}$ mbar) and dried overnight under light vacuum at 19.1 °C. The resulting MAPbI_3_-PCL powder was extruded into a filament using a Filabot EX2. The temperature of the extruder was set between 70 and 80 °C. The final resulting filament was optically black in color with a slight shine. Temperatures above this range would result in a yellow filament indicating degradation of the perovskite crystals.

#### 2.1.2. FDM Perovskite Thin Film Fabrication

A fused deposition modeling system (Lulzbot Mini, Lulzbot Taz 5, or Ultimaker 3) controlled by Cura was used to fabricate all plain and composite filament materials characterized in this work. The nozzle diameter utilized was 2.0 mm. The software was programmed to print a thin-film with thickness measured via digital micrometer after printing. The test pieces were designed in Solidworks and imported into the Cura software as .STL files. The thicknesses tested were 25 μm, 50 μm, 100 μm and 200 μm. Overall length and width of these thin-film samples was 25 mm by 25 mm. A Nikon Digimicro was used to determine the thickness of the FDM printed thin films.

#### 2.1.3. FDM Perovskite Photoconductivity Test Piece

**Figure 2:**
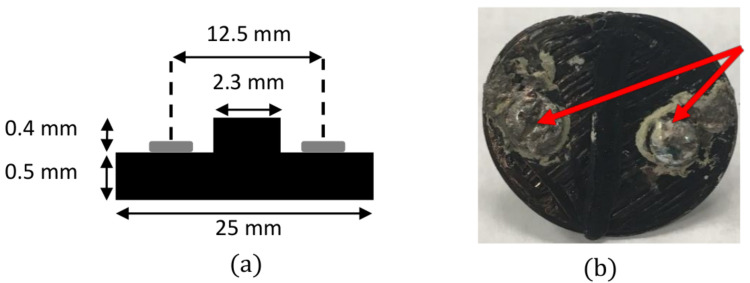
(**a**) Layout of photoconductivity sample. (**b**) Photoconductivity sample and electrode attachment points (red arrow).

Following thin film preparation and evaluation, the MAPbI_3_-PCL composite was printed via FDM into a disk to allow initial investigation of photoconductivity as well as larger-scale 3D printer capability with the new material. The photoconductivity test piece had a diameter of 25 mm and thickness of 0.9 mm. Two circular holes with a diameter of 3 mm, separated by 12.5 mm were printed into the structure as electrode attachment points. Printing was accomplished via commercially available FDM printers. Twin through-bolts were utilized as electrodes and attached to the photoconductivity test piece via MG Chemicals Silver Conductive Epoxy to ensure electrical connection. Figure 2 shows the fabricated test piece and design.

### 2.2. Test Setup

Tests were conducted that included characterization of the fabricated perovskite materials as well as photoconductivity and other operationally-oriented tests. Test setups and characterization procedures are discussed in this section.

#### 2.2.1. Material Characterization Testing

Material characterization of the FDM perovskite material included the use of SEM, UV–Vis (Ultraviolet–visible Spectroscopy), and XRD prior to any material operational characterization. A Hitachi S4800 FE-SEM (Hitachi City: White Plains, NY, USA) was utilized to image the MAPbI_3_ crystals, MAPbI_3_ crystals after coupling agent treatment, and MAPbI_3_-PCL composite film after FDM printing. Specific attention was paid to potential damage caused by the FDM print process as well as action of the coupling agent in the final material.

To more completely establish the successful formation of MAPbI_3_ microcrystals, a Bruker D8 X-ray diffractometer XRD) (Bruker City: Madison, WI, USA) was utilized. This allowed insight into the crystal structure of the perovskite crystals and the printed photoconductivity test pieces (thin films) of MAPbI_3_-PCL. For clarity, XRD spectrum of PbI_2_ powder, MAI powder, MAPbI_3_ powder and PCL-MAPBI_3_ thin film was examined and compared against characteristic peaks associated with MAPbI_3_, and MAPBI_3_-PCL to validate integration and processing into the desired perovskite filament.

#### 2.2.2. Absorbance and Transmittance/Reflectance Testing of PCL Thin Films

A Jasco V-530 UV–Vis spectrophotometer (Jasco Inc., Easton, MD, USA) was utilized to characterize the absorbance spectrum of PCL and MAPbI_3_-PCL thin-films. For PCL and MAPBI_3_-PCL the thin films, absorbance was monitored across a range of about 400–1050 nm. The range was selected based on prior work in the field that indicated MAPBl_3_ absorbance peaking in this range [24,25,26]. This was done for each thin film sample, with particular attention to the effects of perovskite and its addition into the test materials.

Transmittance spectrum was also investigated. A Filmetrics RT-10 refractometer (Filmetrics, Roselle, CA USA) was utilized to determine the transmittance and reflectance characteristics of the various FDM printed thin-films. The fabricated thin-film pieces (25 μm, 50 μm, 100 μm, and 200 μm thick) were investigated to assess transmittance through polycaprolactone as well as transmittance vs. thickness levels and light allowance into active layers.

Based on those tests, 200 μm film was tested further including tests at room temperature and after heating (40 °C, 5 min) to monitor performance changes. In addition, tests were also conducted on PCL, MAPbI_3_-PCL, and samples of MAPbI_3_-PCL-LICA 38 (coupling agent).

#### 2.2.3. I-V Sweep and Photocurrent Testing

Using the FDM perovskite test pieces constructed for the purpose, electrodes were attached via wire to a Keithley 2400 Sourcemeter(Keithley/Tektronix, Beaverton, OR, USA). Current–voltage (I-V) sweeps from −10 V to 10 V by 0.1 V were performed to determine current increase once the material was exposed to light. Dark conditions were also tested in order to provide conclusive results indicating the material reacted to light and not I-V sweep.

All photoconductivity tests were conducted at room temperature. The outcome of these tests was compared to validate perovskite absorption ranges based on prior work in the field [24,25,26]. The light was applied with a Spectra-Physics 66900 solar simulator (Newport/Spectra-Physics, Milpitas, CA, USA). Input power was varied from 50–80 W, which corresponded to an incident flux of 252–590 W/m${}^{2}$. Flux was measured with a Newport 91150V Reference Cell and Meter (Newport, Milpitas, CA, USA). This product followed ISO-17025 standards and the output reading was in sun units (where 1 sun is equal to 1000 W/m${}^{2}$ at 25 °C and Air Mass 1.5 Global Reference). This corresponded to approximately half of what could be expected on a sunny, summer day (approximately 1000 W/m${}^{2}$). This provided an adequate but conservative simulated solar input for these tests and investigated how light intensity affected photoconductivity. Incident fluxes at each input power level are summarized in Table 1.

## 3. Results and Discussion

This section presents the specific results of the various tests that were conducted on both material and full solar cell constructs. Specific material test results are presented first, followed by the solar photo-conductivity test piece performance test results.

### 3.1. Material Characterization Test Results

**Figure 3:**
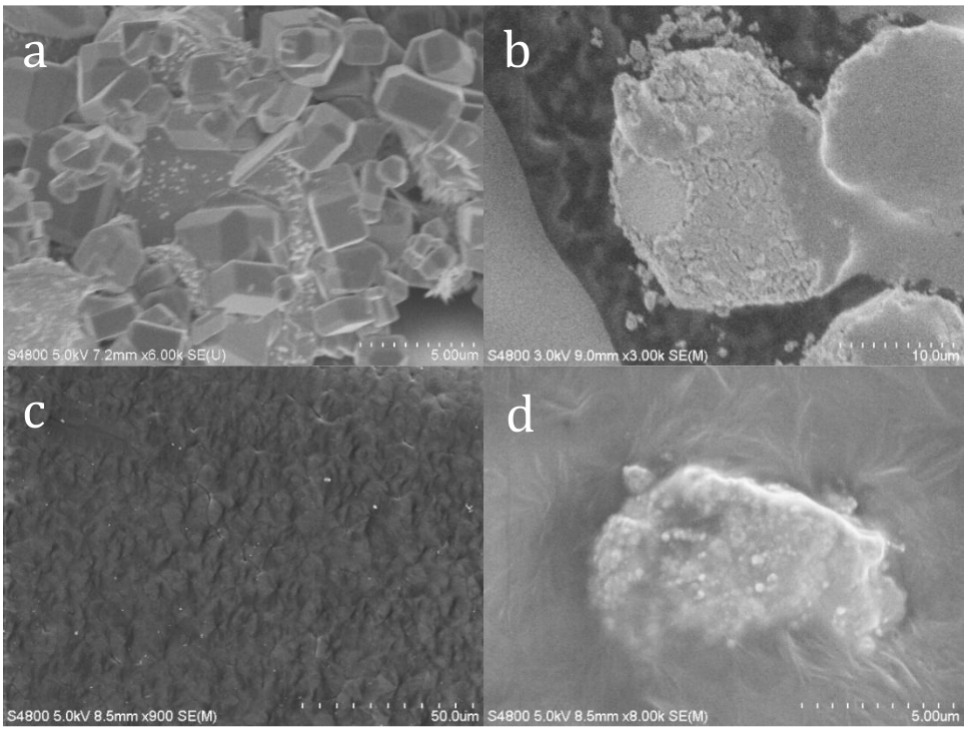
SEM images of (**a**) perovskite (**b**) coupling agent treated perovskite (**c**) PCL-50 wt.% Perovskite (**d**) PCL-50 wt.% Perovskite.

Figure 3 shows SEM images of perovskite, perovskite treated with coupling agent, FDM printed MAPbI_3_-PCL thin-film, and PCL/perovskite interface. Figure 3a shows the perovskite crystals, which had a cubic structure before coupling agent introduction. The different size of crystals remained following fabrication as well. Figure 3b shows the perovskite crystals after coupling agent introduction. As can be noted in the image, the cubic structure remained, however it was coated with the silane coupling agent used in the study. After printing the MAPbI_3_-PCL thin-film, the surface morphology was rough and had the appearance of embedded cubes as depicted in Figure 3c. The coupling agent aided in creating a voidless interface between the perovskite and PCL shown in Figure 3d.

This investigation via SEM images showed that coupling agent introduction, filament extrusion, and FDM printing process did not damage the structure of the MAPbI_3_ crystals. Thus, the FDM filament manufacture and printing process was found to be a viable option to produce MAPbI_3_ composites.

**Figure 4:**
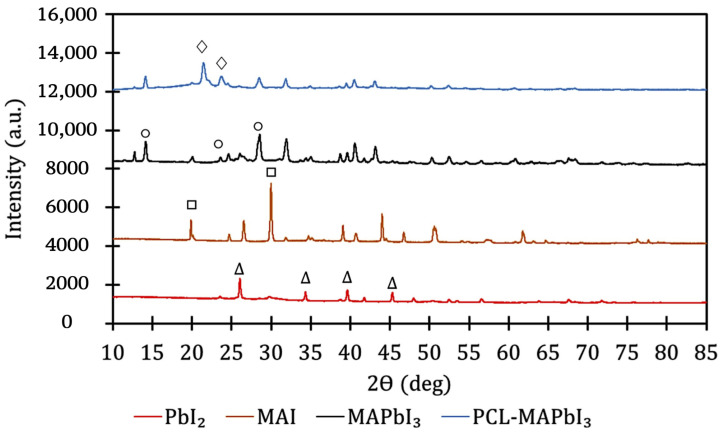
XRD of PbI_2_, MAI, MAPbI_3_, and MAPbI_3_-PCL.

**Figure 5:**
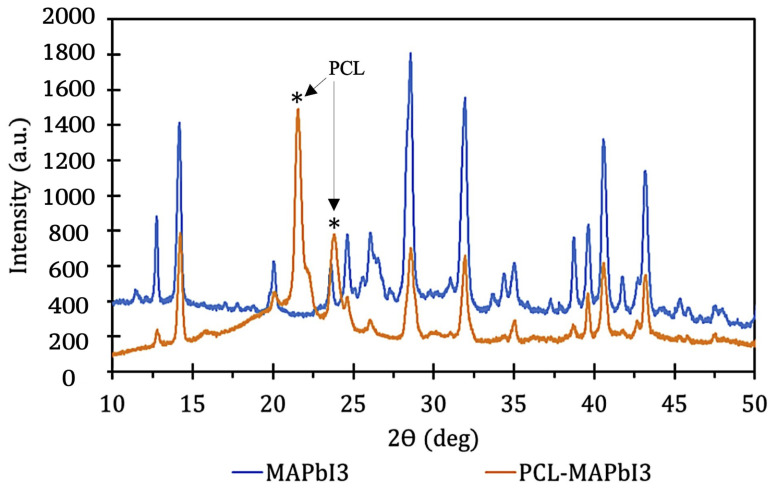
XRD of MAPbI_3_ and PCL-MAPbI_3_. Note: ∗ indicates PCL corresponding lattice plane peaks.

XRD characterization was performed and is presented in Figure 4 and Figure 5. Table 2 summarizes the findings. In general, these findings indicated the successful formation of MAPbI_3_ microcrystals, integration of the MAPbI_3_ microcrystals into the PCL matrix, and good stability with lack of degradation during the FDM printing process.

Figure 4 shows XRD spectrum of PbI_2_ powder, MAI powder, MAPbI_3_ powder, and PCL-MAPBI_3_ thin film. The diffraction peaks at 2Θ= 26${}^{\circ }$, 34${}^{\circ }$, 39${}^{\circ }$, 46${}^{\circ }$ (denoted with Δ) correspond to the (101), (102), (110), and (103) lattice planes of PbI_2_ [27]. Peaks at 2Θ= 20${}^{\circ }$ and 30${}^{\circ }$ (denoted with □) correspond to the (002) and (003) lattice planes of MAI [28]. MAPbI_3_ contained peaks at 2Θ = 14${}^{\circ }$, 23${}^{\circ }$, 28.3${}^{\circ }$ and 28.6${}^{\circ }$ (denoted with ∘), which corresponded to the (002), (121), (004), and (220) lattice planes [8]. The characteristic peaks associated with MAPbI_3_ remained in the PCL-MAPbI_3_ composite.

Figure 5 shows an XRD spectrum of perovskite overlaid on the spectrum for PCL-MAPbI_3_ composite. Although the peak intensities were reduced in the PCL-MAPbI_3_ composite, the MAPbI_3_ peaks show the integration and processing of the MAPbI_3_ into PCL-MAPbI_3_ filament. The peaks at 2Θ = 21${}^{\circ }$ and 23${}^{\circ }$ (denoted with ∗ in Figure 4) peaks corresponded to the (110) and (200) lattice planes of PCL and agree with literature values [29,30,31,32].

One key goal of these essential material evaluations was to confirm that the perovskite material itself did not exhibit significant degradation either in the preliminary mixing, or subsequent processing as it was made suitable for FDM utilization as a ‘printable’ polymer. This was shown through both SEM and XRD analysis where expected perovskite properties were evident throughout. This allowed continued investigation of the polymer in more optically-oriented testing.

### 3.2. Absorbance and Transmittance Spectrum Results

**Figure 6:**
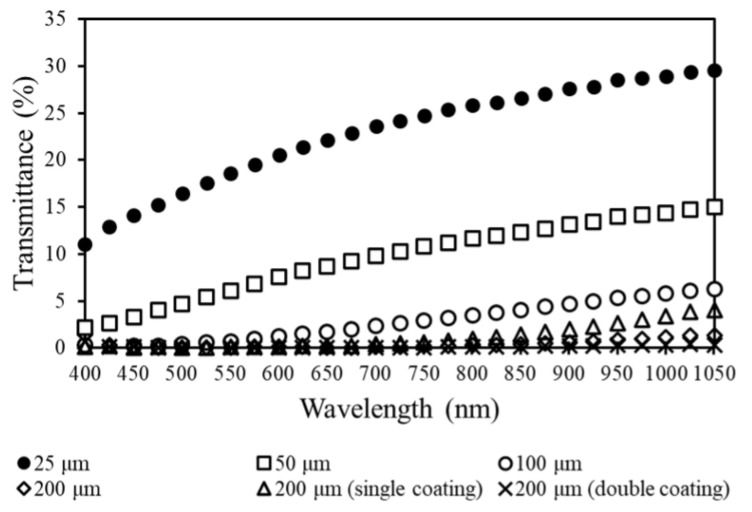
Transmittance spectrums for PCL thin films.

With material properties verified, evaluation turned to optical performance and properties. Figure 6 shows the transmittance spectrum for PCL thin films from 400–1050 nm. The thicknesses tested were 25 μm, 50 μm, 100 μm, and 200 μm. The 200 μm thicknesses were also tested with one or two coatings of Smooth-On XTC-3D. The maximum transmittance occurred in the 25 μm sample, which had a transmittance of 29.6% at 1050 nm. Samples had decreasing transmittance with increasing thickness, the lowest transmittance occurred in the 200 μm sample with two coatings of the Smooth-On XTC-3D at 0.9% and 393 nm. Maximum transmittance did increase from a single coating of Smooth-On XTC-3D on a 200 μm PCL sample to 4.1% when compared to the 200 μm plain PCL sample, which attained a maximum transmittance of 1.4%. Transmittance increased for all samples with increasing wavelength except the 200 μm sample with a double coating of Smooth-On XTC 3D.

This demonstrated the effect of thickness on optical transparency with thinner FDM-printed samples allowing more light into the active layer in a solar cell structure. However, even at a thickness of 200 μm light still entered the active layer. This also confirmed that PCL allowed transmission of optical wavelengths absorbed by MAPbI_3_ to transmit through the layer.

The use and performance of the XTC-3D coating is also worth noting. The primary purpose of the coating was to reduce surface roughness, which reduces light scattering effects of the 3D printed film. However, while one layer improved performance, a second layer had negative effects. One obvious reason is that increased layering effectively reduced transmittance simply via thickness, despite the initial benefit of reduced light scatter. Further, the use of a second layer may cause negative light-transmittance reflecting effects. For instance, the second layer of the XTC-3D may produce an XTC–air–XTC interface to form causing light to bounce back and forth. This is an outcome of the tendency of XTC-3D to bond to itself in layering processes. Consequently, this would lead to the longer wavelength range being absent in the transmittance spectrum of the double-coated samples.

**Figure 7:**
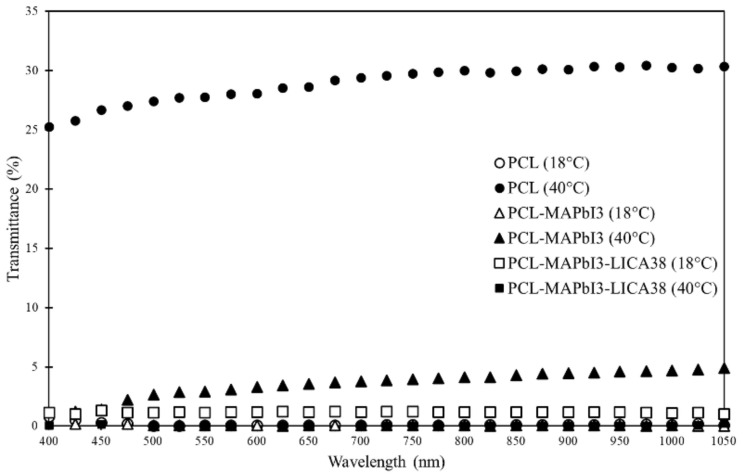
Transmittance spectrum of PCL, PCL-MAPbI_3_, and PCL-MAPbI_3_-LICA38.

Figure 7 shows the transmittance spectrum for 200 μm samples of PCL, PCL-MAPbI_3_, and samples of PCL-MAPbI_3_-LICA38 at room temperature and after heating at 40 ${}^{\circ }$C for 5 min. Transmittance generally increased for all samples as wavelength increased. Additionally, transmittance increased once the samples were heated except in the case of the PCL-MAPbI_3_-LICA38, which decreased in transmittance after heating. The maximum transmittance occurred in the PCL sample after heating at 30.4%. The addition of MAPbI_3_ into PCL reduced the maximum transmittance to 4.9% after heating the sample. While initially seemingly a negative outcome, this reduction in transmittance could be a good indicator of absorbance due to the presence of MAPbI_3_ micro/nanocrystals.

**Figure 8:**
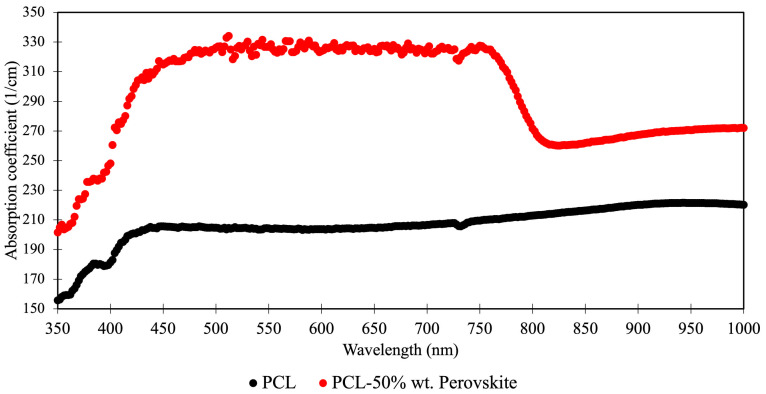
UV–Vis of PCL thin-film and PCL-MAPbI_3_ thin-film.

Figure 8 shows the UV–Vis absorbance spectrum of a plain PCL thin-film and a MAPbI_3_-PCL thin-film from 350–1000 nm. After the addition of perovskite, the absorbance across all wavelengths was increased. The most prominent increases occurring from 400–800 nm. Given this known absorption range from prior work in the field, this confirmed an active and receptive perovskite material [24,25,26].

### 3.3. Photocurrent Characterization Results

**Figure 9:**
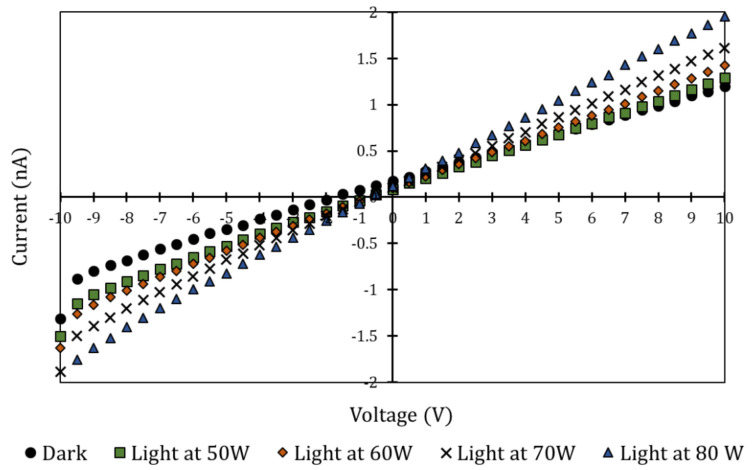
I-V curve of MAPbI_3_-PCL composite in dark, 50 W, 60 W, 70 W, and 80 W.

Following setup and test procedure described in Section 2, photocurrent characterization tests were completed on the fabricated test piece with attached electrodes. Figure 9 shows I-V curve test result of the photoconductivity of the MAPbI_3_-PCL composite at different power input solar simulator levels. These ranged from 50–80 W. The linear current–voltage curve indicated ohmic contacts were formed at the composite/electrode interfaces. In dark conditions, the resistance was 9.79 $\times \;10^{9}$ ohms. At 80 W, the resistance was 5.46 $\times \;10^{9}$ ohms. It is interesting to note that resistance was decreased as lighting intensity was increased (at the same voltage levels). This signified an increase in photocurrent at higher light intensities, which indicated increased electron–hole pair generation. Table 3 further summarizes the results.

In summary, reaction of the new polymer FDM materials showed the promise of an active solar layer even given the fabrication steps that included FDM extrusion. Photo-reaction performance of the material in the thin layer FDM pieces and 3D fabricated photo conductivity test pieces both showed solar absorption capability and electron–hole pair generation. Further, heating the material increased transmission and performance which holds promise for real devices that operate at more elevated temperatures.

## 4. Conclusions

Perovskite solar cells have generated major research interest since their discovery and offer beneficial properties to solar cell fabrication like long minority charge carrier distance, high light absorption, and simple fabrication methods. Despite the potential for these solar cells, however, stability remains an issue due to material degradation in UV light and other common atmospheric elements. This work investigates the potential for fused deposition modeling (FDM), or 3D printing, to produce perovskite solar cells that can address some of these challenges. Many of these are achieved specifically through the encapsulation and printing process of FDM.

Specific steps have been demonstrated to produce the printable polymer material including MAPbI_3_ microcrystal fabrication in ambient atmosphere, integration of MAPbI_3_ into a polymer matrix without degradation, and usage of that MAPbI_3_-PCL composite to fabricate a solar active material. Outcome of testing included a noted increasing transparency with increasing temperature and hence, improving conductivity when exposed to simulated solar light. Further, increased electron-hole pair generation was observed for 200 μm FDM-printed PCL film, achieving a 45% reduction in resistance under peak incident flux of 590 W/m${}^{2}$ with the addition of MAPbl_3_.

While these tests were conducted at relatively conservative simulated solar loadings, additional testing at higher solar loading is planned for future work alongside construction of a full solar stack. Fused deposition modeling establishes opportunity to integrate aligned conductive fillers into this composite to improve charge extraction and power output via integrated electrodes. This demonstrates the feasibility and potential of FDM in solar cell fabrication.

## Figures and Tables

**Table 1:** Incident flux versus input power.

Input Power (W)	Incident Flux (W/m${}^{2}$)
50 W	252 W/m${}^{2}$
60 W	364 W/m${}^{2}$
70 W	459 W/m${}^{2}$
80 W	590 W/m${}^{2}$

**Table 2:** Summary of diffraction peaks and lattice planes for PbI_2_, MAI, MAPbI_3_, and PCL-MAPbI_3_.

Material	Diffraction Peak 2Θ	Lattice Plane (hkl)	Reference
Lead Iodide (PbI_2_)	26${}^{\circ }$	(101)	[27]
	34${}^{\circ }$	(102)	
	39${}^{\circ }$	(110)	
	46${}^{\circ }$	(103)	
Methylammonium	20${}^{\circ }$	(002)	[28]
Iodide (MAI)	30${}^{\circ }$	(003)	
Methylammonium Lead	14${}^{\circ }$	(002)	[12]
Triiodide (MAPbI_3_)	23${}^{\circ }$	(121)	
	28.3${}^{\circ }$/28.6${}^{\circ }$	(004)/(220)	
Polycaprolactone	21${}^{\circ }$	(110)	[33,34]
Methylammonium Lead	23${}^{\circ }$	(200)	
Triiodide (PCL-MAPbI_3_)			

**Table 3:** Resistances of photoconductivity test piece at different power levels.

Input Power (W)	Resistance (M-ohms)
Dark, 0 W	9.79 $\times \;10^{3}$
50 W	8.29$\;\times \;10^{3}$
60 W	7.58$\;\times \;10^{3}$
70 W	6.62$\;\times \;10^{3}$
80 W	5.46$\;\times \;10^{3}$

## Data Availability

Data archived and maintained by authors.
